# Nanotransethosomes for enhanced transdermal delivery of mangiferin against rheumatoid arthritis: formulation, characterization, invivo pharmacokinetic and pharmacodynamic evaluation

**DOI:** 10.1080/10717544.2023.2173338

**Published:** 2023-02-02

**Authors:** Syeda Nashvia Adin, Isha Gupta, Md Abdur Rashid, Yahya Alhamhoom, Mohd Aqil, Mohd Mujeeb

**Affiliations:** a Phytomedicine Laboratory, Department of Pharmacognosy & Phytochemistry, School of Pharmaceutical Education & Research, Jamia Hamdard University, New Delhi, India; b Department of Pharmaceutics, College of Pharmacy, King Khalid University, Abha, Saudi Arabia; c Department of Pharmaceutics, School of Pharmaceutical Education & Research, Jamia Hamdard University, New Delhi, India

**Keywords:** Mangiferin, transethosome, rheumatoid arthritis, Box-Behnken design, transdermal delivery, modified nanoliposomes

## Abstract

The present research study limns the preparation of MNF loaded transethosomes (MNF-TE) to improve MNF solubility, bioavailability and permeation through skin layers for transdermal delivery. MNF-TE was formulated using thin-film hydration method and optimization was done using Box-Behnken design (BBD). MNF-TEopt was characterized for Polydispersity index (PDI), vesicle size, entrapment efficiency, zeta potential and in vitro MNF release. For further evaluation, Pharmacokinetic study, Transmission electron microscopy (TEM), Skin permeation study and Confocal scanning laser microscopy (CLSM) were performed withal. The MNF-TEopt presented spherical and sealed shape vesicles with small vesicle size of 148.6 nm, entrapment efficiency of 74.23%, PDI of 0.1139 and in vitro release of 65.32%. The CLSM study unveiled that the developed formulation has greater permeation of MNF across the skin layers in comparison with the MNF suspension gel. The pharmacokinetic study demonstrated C_max_ and AUC_0-24 h_ of 6.94 ± 0.51 μg/ml and 43.92 ± 7.90 μg.h/ml via transdermal route in comparison to C_max_ and AUC_0-24 h_ of 3.74 ± 1.91 μg/ml and 22.96 ± 9.76 μg.h/ml presented by MNF-TE oral administration. The in vivo study revealed that the MNF-TE gel has good anti-arthritic potential in comparison with the standard diclofenac gel which was evinced by radiographic analysis and histopathological studies. Further, skin irritation study on Wistar albino rats confirm that the developed MNF-TE formulation is safer for skin application. The current investigation corroborated that the prepared TE vesicle formulation is a treasured carrier for the MNF transdermal delivery for the management of rheumatoid arthritis.

## 1. Introduction

Rheumatoid arthritis (RA) is a chronic autoimmune-mediated systemic disorder characterized by nonspecific peripheral joint inflammation, pannus formation, hyper proliferation of synoviocytes, bone erosion, angiogenesis, chronic pain and cartilage degradation (Liu et al., 2017). RA is a preeminent cause of morbidity and deformity, depicting worldwide prevalence betwixt 0.3 to 1% (Almoallim et al., 2021).

The current treatment strategies for RA include Biologicals, Disease-modifying anti-rheumatic drugs (DMARDs), Corticosteroids and NSAIDS. Howbeit, these treatments at a higher dose and on prolonged use are accompanied with adverse effects such as gastrointestinal and hepatic disorders, humoral disturbances, and immunodeficiency and are even costly and inconvenient (Liu et al., 2017). Thereby, the use of natural phytoconstituents have acquired increased attention owing to its immunomodulatory properties, flair for inflammatory disorders and withal devoid of infelicitous effects manifested by synthetic drugs (Hughes et al., 2017).

Mangiferin (MNF) is a natural xanthanoid chiefly present in *Mangifera indica, Iris unguicularis, Salacia chinensis, Cyclopia genistoides, Anemarrhena asphodeloides*, and *Bombax ceiba* (Khare et al., 2016; Tang et al., 2014). MNF has been copiously used in myriads of therapeutic fields because of its profuse pharmacological profile including antidiabetic, antibacterial, antiarthritic, antitumor, antiviral, anti-inflammatory, antitumor, immunomodulatory, lipometabolism regulating, hepatoprotective, cardioprotective, anti hyperuricemic, antiaging, antioxidative, neuroprotective, antipyretic, and analgesic activity MNF evince promising antiarthritic potential by virtue of its anti-oxidant, anti-inflammatory and analgesic property (Adin et al., 2022a, 2022b, 2022c, 2022d; Adin et al., 2023; Gupta et al., 2022a, 2022b, 2022c, 2022d, 2022e). Luczkiewicz et. al adumbrated that MNF acts by showing pro-apoptotic effect toward Fibroblast-like synovial (FLS) cells and anti-inflammatory potential by modulating NF-κB pathway and pro-inflammatory cytokines down-regulation (Luczkiewicz et al., 2014).

Despite of its meritorious therapeutic potential, its poor aqueous solubility (111 μg/ml in water) and low oral bioavailability (1.2%) circumscribe its clinical use (Han et al., 2010; Bhattacharyya et al., 2014; Wang et al., 2014). Ergo, transdermal delivery of MNF is an alluring alternative in this scenario. Pharmaceutical researchers are enthralled by transdermal delivery system as it is endowed with prowess of non-invasiveness, low toxicity and surpassing hepatic metabolism. Howbeit, MNF passive permeation through the skin is hampered by its physicochemical properties and skin barrier (Qindeel et al., 2020; Tekko et al., 2020).

To vanquish such barrier various vesicular nanocarriers have been developed. Wei et. al successfully prepared mangiferin-loaded liposomes (Wei et al., 2014). However, the stratum corneum limits the permeation of conventional liposomes to the upper skin layer with minimal permeation to the deeper skin layers (Touitou et al., 2000). Ethanol and surfactants can be added to conventional liposomes to increase deformability of the lipid membrane which inturn results in enhanced drug permeation across the stratum corneum (Cevc, 1996; El Maghraby et al., 2004; Carita et al., 2018) . Mir-palomo et.al have developed ultradeformable vesicles of mangiferin for improved cutaneous delivery (Mir-Palomo et al., 2016). Abd El Alim et. al conducted a comparative study to assess the efficacy extent of skin permeation of diflunisal (DIF) loaded transferosomes, ethosomes and liposomes invivo and invitro and found that transferosomes and ethosomes showed enhanced skin permeation and higher drug encapsulation efficiency of DIF (Carita et al., 2018).

The transethosome, a novel nanovesicle carrier are pliable systems consisting a blend of ethanol, surfactant and lipid. It combines the properties of transferosome and ethosome which in turn gives better biocompatibility, increased vesicle mobility and higher encapsulation rate in comparison with transferosome and ethosome (Song et al., 2012; Abd El-Alim et al., 2019). Drug permeation via skin is improved by ethanol as it disrupts the packaging of compact lipid stratum corneum forbye. So, it is evident that transethosomes are paragon carrier for transdermal delivery (Abdulbaqi et al., 2016).

The intent of the study is to develop mangiferin-loaded transethosomes (MNF-TE) by thin-film hydration method using phospholipon 90 G, ethanol and sodium cholate. For the optimization of developed formulation, Box-Behnken design (BBD) was employed by taking phospholipon 90 G, ethanol and sodium cholate as independent variables and their impact were assessed on dependent variables viz. polydispersity index (PDI), vesicle size and entrapment efficiency (EE). Further, the optimized formulation (MNF-TEopt) was remolded into carbapol-934 P based gel and evaluated for confocal scanning laser microscopy (CLSM), skin permeation study, invitro-release kinetics, invivo pharmacokinetic, pharmacodynamics and skin-irritation studies.

## 2. Materials and methods

MNF standard was acquired from Sigma Aldrich (India), MNF API was procured from Yucca enterprises (India), Triethanolamine was procured from Fischer Scientific (Mumbai, India). Phospholipon 90 G was procured from Lipoid (Germany), Sodium cholate was procured from DC Fine Chemicals (India), Polyethylene glycol-400, Methanol, Chloroform and carbapol 934 P were acquired from SD fine chemicals (Mumbai, India). ELISA kits for inflammatory cytokines were acquired from ABclonal technology Co., Ltd. (India). Freund’s adjuvant, Complete cell suspension was procured from Sigma Aldrich (India). All HPLC solvents were acquired from SD Fine chemicals (Mumbai, India).

### 2.1. Formulation of mangiferin loaded transethosomes (MNF-TE)

The mangiferin loaded transethosomes were developed using thin film hydration technique. In a round bottomed flask (RBF), defined quantity of Phospholipon 90 G (lipid), Mangiferin (drug- 1 mg/ml) and sodium cholate were dissolved in methanol: chloroform (3:1, v/v). The organic blend was evaporated under vacuum (low pressure) at 60 °C to attain a thin uniform layer of lipid deposited around the walls of RBF using rotary evaporator. The RBF was placed in desicator for 24 hours. The dried film was hydrated using solution of water: ethanol (7:3) for 1 hour and stowed in refrigerator to acquire adequate swelling. The attained dispersion was probe sonicated for 4 minutes continuously at 4 °C using titanium probe ultra sonicator (UP 100 H, Hielscher Ultrasonics GmbH, Berlin) and further characterized for PDI, entrapment efficiency, zeta potential and vesicle size (Moolakkadath et al., 2018).

### 2.2. Optimization of transethosomes using Box-Behnken design (BBD)

Preliminary screening trials were done to ascertain the potential parameters that impact the beneficial properties of TE for transdermal delivery. After the determination of desirable parameters, a three-factor BBD was employed using design expert version 13 software (State-ease, USA). BBD was employed to ascertain the impact of lipid concentration, sodium cholate concentration and ethanol concentration on response variables- PDI, entrapment efficiency and vesicle size. The design comprised of 17 experimental runs (Table 2). Quadratic response surface model (generated by the Box-Behnken design) was as follows:

$${Z=k}_{0}{+k}_{1}X_{1}{+k}_{2}X_{2+}k_{3}X_{3+}k_{12}X_{1}X_{2+}k_{13}X_{1}X_{3+}k_{23}X_{2}X_{3+}k_{11}X_{1}{}^{2}{+k}_{22}X_{2}{}^{2}{+k}_{33}X_{3}{}^{2}$$

**Table 2.** Box-Behnken experimental design with measured responses.

Formulation	Independent variables			Dependent variables		
	A	B	C	Y1	Y2	Y3
1	50	30	30	0.1468 ± 0.007	117.12 ± 4.09	58.2 ± 1.34
2	50	20	20	0.167 ± 0.003	176.96 ± 3.92	80.45 ± 1.68
3	60	30	20	0.1137 ± 0.009	148.2 ± 2.98	74.39 ± 2.09
4	60	30	20	0.1135 ± 0.009	148.9 ± 2.98	74.51 ± 2.09
5	60	30	20	0.1139 ± 0.009	148.6 ± 2.98	74.23 ± 2.09
6	60	30	20	0.1147 ± 0.009	147.9 ± 2.98	74.11 ± 2.09
7	70	30	30	0.1265 ± 0.003	140.17 ± 4.03	64.22 ± 2.98
8	70	30	10	0.2752 ± 0.012	187.34 ± 4.26	81.55 ± 1.62
9	50	40	20	0.1442 ± 0.005	110.65 ± 4.36	55.21 ± 1.72
10	60	40	10	0.1191 ± 0.003	144.9 ± 4.67	72.11 ± 1.87
11	60	30	20	0.1123 ± 0.009	148.28 ± 2.98	74.98 ± 2.09
12	60	20	30	0.1237 ± 0.004	162.25 ± 4.23	73.23 ± 1.62
13	70	20	20	0.2818 ± 0.012	198.51 ± 2.98	80.1 ± 1.92
14	60	40	30	0.0653 ± 0.002	96.74 ± 2.09	44.14 ± 1.76
15	70	40	20	0.1618 ± 0.004	132.21 ± 2.32	67.98 ± 1.23
16	50	30	10	0.1209 ± 0.008	171.18 ± 3.07	73.22 ± 2.09
17	60	20	10	0.1977 ± 0.007	213.01 ± 4.35	81.26 ± 1.98
**Quadratic model**		**R^2^**	**Adjusted R^2^**	**Predicted R^2^**	**S.D.**	**%CV**
**Response (Y_1_)**		0.9998	0.9995	0.9976	0.0012	0.8397
**Response (Y_2_)**		0.9998	0.9995	0.9970	0.6853	0.4493
**Response (Y_3_)**		0.9984	0.9963	0.9780	0.6187	0.8737

Wherein, Z - Predicted response

X_i_ - the independent variables

k_i_, k_i2_, k_i3_ – quadratic, linear and interactive coefficients

The selected independent variables were Phospholipon 90 G concentration (X_1_), sodium cholate concentration (X_3_) and Ethanol concentration (X_2_) and the dependent variables were PDI (Y_1_), vesicle size (Y_2_) and EE (Y_3_) (Table 1).

**Table 1.** Variables and their levels.

	Levels		
Variables	Low (-1)	Medium (0)	High (+1)
Independent Variables			
A: Phospholipon 90G (mg)	50	60	70
B: Ethanol (%)	20	30	40
C: Sodium cholate (mg)	10	20	30
Dependent Variable			
Y_1_: PDI Y_2_: Vesicle size (nm) Y_3_: Entrapment efficiency (%)			

### 2.3. Characterization of MNF-TEopt

#### 2.3.1. Vesicle size, zeta potential and polydispersity index (PDI)

The vesicle size, PDI and zeta potential of developed MNF-TE were estimated using zetasizer (Malvern instruments, Wancestershine, UK) after diluting the sample with Mill-Q water (50 times) at 25 ± 1 °C in triplicate at a scattering angle of 90° C (Moolakkadath et al., 2018).

#### 2.3.2. Entrapment efficiency

Ultracentrifugation method was employed to assess the entrapment efficiency of MNF-TE (Moolakkadath et al., 2018). The samples were stowed overnight at 4 °C and then subjected to centrifugation for 1 hour at 4 °C using centrifuge at 20,000 rpm (REMI, cooling centrifuge, Mumbai, India). The filtrate comprising free mangiferin was seperated and diluted with the appropriate medium and further the mangiferin content was assessed using HPLC. The entrapment efficiency was determined using formula:

$$\frac{\%\;\text{EE}=\text{Total}\;\text{mangiferin}-\;\text{Mangiferin}\;\text{in}\;\text{supernatant}}{\text{Total}\;\text{mangiferin}}\times 100$$

The mangiferin content was assessed by HPLC analysis with mobile phase water: acetonitrile (0.05% orthophosphoric acid) (85:15) at a 1 ml/min flow rate using C_18_ column. UV detection wavelength was fixed at 258 nm (Adin et al., 2022a).

### 2.4. Transethosomes morphology

The morphology analysis of the MNF-TEopt was carried out using transmission electron microscopy (TEM- Tecnai, CM 200, Philips scientific, NY). Before analysis, on copper grid, a drop of diluted sample was applied and then analyzed under transmission electron microscope after negatively staining with phosphotungstic acid (2%) (Moolakkadath et al., 2018).

### 2.5. Formulation of mangiferin-loaded transethosomal gel

The MNF-TEopt was transformed into a transdermal gel to protract the retention of formulation on rat skin for longer period of time. The gel was prepared by taking defined amount of carbapol-934 P (1% w/w) in distilled water and left overnight to swell. Later, triethanolamine (for pH- modification), chlorocresol (0.1% as preservative) and 15% w/w polyethylene glycol 400 (as plasticizer) were added and then optimized mangiferin-loaded transethosome was added to attain homogenous gel mixture dropwise with continuous homogenization (Moolakkadath et al., 2018).

### 2.6. Texture analysis

TA-XT plus texture analyzer (Stable Micro system, UK) was employed to evaluate the texture analysis of MNF-TEopt gel based on different parameters like firmness, work of cohesion, adhesiveness and cohesiveness. The study was carried out in a simple compression mode by taking MNF-TEopt gel in a glass beaker (50 ml). The test speed of 2.0 mm/s was used and distance traveled by probe was 10,000 mm. The post-test speed of 2.0 mm/s was used to bring back the probe. The auto trigger force of 10.0 g was confronted by the upper probe on contact with the gel. Texture exponent 32 software was used to analyze the force requisite to disengage the probe from gel (Moolakkadath et al., 2018).

### 2.7. In-vitro MNF release study

Drug release dialysis membrane technique was adopted to evaluate the in-vitro release of MNF suspension gel (control) and MNF-TEopt gel. Both formulations (1 mg/g) were thronged in the 12000–14000 Da preactivated dialysis membrane (Hi Media, Mumbai) which was affixed with the shafts placed in a release medium of phosphate buffer saline (pH 6.8) contained in a beaker (500 ml), regulated at a temperature of 37 ± 2° C and a uniform stirring of 100 rpm. At preordained time intervals of 0.5, 1, 2, 4, 8 and 12 hours, samples were collected and replenished with fresh release medium. The mangiferin content was quantified using RP-HPLC method and the graph was plotted betwixt time (hours) and % cumulative drug release (Moolakkadath et al., 2018).

### 2.8. Skin permeation study

The skin permeation of MNF-TEopt and MNF suspension gel (control) were assessed using Franz-diffusion cell. Franz-diffusion cell of effective permeation area of 1.0 cm^2^ and receiving cell volume of 15 ml was used. The rat skin was prepared and affixed on the receiver compartment confronting upward stratum corneum side. The release media was thronged in the receiver cell and MNF-TEopt gel in the donor cell. The constant temperature of 37 ± 2° C for 24 hours with constant stirring at 150 rpm was maintained. At preordained time intervals of 0.5,1 2, 4, 8 and 12 hours, samples were collected and replenished with fresh release medium and mangiferin content was quantified using RP-HPLC method (Moolakkadath et al., 2018).

### 2.9. Confocal laser scanning microscopy (CLSM)

The MNF suspension with rhodamine B dye (control) and rhodamine B dye loaded transethosomal formulation were applied to the excised rat abdominal skin for 6 hours at 37 °C mounted on Franz diffusion cells. After 6 hours, to remove the excess amount of dye, skin samples were cleansed with distilled water. Then the skin samples were mounted on the glass slide and sliced into small sections of 6-10 um thickness. Then the slide fronting upper stratum corneum was espied under CLSM (Leica TC SPE-1lw, Leica microsystem, Germany) employing argon laser beam (emission at 570 nm & excitation at 488 nm) (Moolakkadath et al., 2018).

### 2.10. Skin irritation study

To determine skin irritation of MNF-TEopt gel in rats, Draize score test was employed. Six wistar albino rats in group of 3 were divided into 2 groups to check the skin irritation of MNF-TEopt gel: Group I was treated with the formalin solution (10%) and Group II was treated with the MNF-TEopt gel. The vicinal untreated area of rats was used as control skin and the edema and erythema scores were estimated. The formulation was applied to the rat skin, and to assess the edema and erythema, visual scoring was carried out after the sample removal (Touitou et al., 2000).

### 2.11. Pharmacokinetic study

Wistar albino rats (100-150 g) were selected and housed into group of 3 per cage in 12 h-light dark cycle at 25 ± 2° C after approval from Institutional Animal Ethics Committee (IAEC) of Jamia Hamdard (Protocol No. 1847). Nine wistar albino rats in group of 3 were distributed into 3 groups: Group I as control, Group II treated with MNF-TE oral formulation, and Group III treated with MNF-TE gel transdermally. The rats were anesthetized using thiopentone sodium and at 0 (pre-dose), 4, 8 and 24 hours; 0.2 ml of blood was collected from tail vein of rats in EDTA-containing tube. Tubes were stowed for 30 minutes at room temperature and then the plasma was separated from blood through centrifugation (10,000 rpm, 10 minutes). The plasma samples were separated and stowed at −20° C and mangiferin content was analyzed using RP-HPLC method and C_max_ and AUC was calculated (Zhang et al., 2021).

### 2.12. Anti-arthritic activity

Wistar albino rats (100-150 g) were acquired from Central Animal House Facility, Jamia Hamdard, New Delhi, India and kept under standard laboratory conditions (12 h-light dark cycle, 25 ± 2^0^ C) after approval from Animal ethics committee (Jamia Hamdard). The study was carried out to determine the anti-arthritic and analgesic activity of MNF-TE gel. The rats in group of 6 were distributed into four groups: Group P was treated as negative control (toxic group), Group Q was treated with standard diclofenac gel, Group R was treated with MNF-TE gel and Group S was treated as Positive control which received control gel formulation.

#### 2.12.1. In vitro anti-arthritic activity

Protein denaturation method was employed to assess the in vitro antiarthritic activity of MNF-TEopt gel and marketed diclofenac gel formulation (Abidin et al., 2016). The mechanism involves alteration in disulfide bonding and electrostatic hydrogen during denaturation. The 0.5 ml of reaction mixture was prepared by taking 5% aqueous solution of Bovine serum albumin (4.5 ml) and formulation (0.5 g) and then pH was adjusted to 6.3 by addition of 0.1 N HCl. Further, samples were incubated at 37 ° C for 20 min and then heated for 30 min at 60 ^0 ^C. Then after cooling the sample, PBS of pH 6.3 (2.5 ml) was added to each sample. Similarly, control solution was prepared and using UV spectrophotometer, turbidity was measured at 600 nm. The percentage inhibition was determined using formula:

$$\frac{\text{Percentage}\;\text{inhibition}=100-\;\left(\text{Absorbance}\;\text{of}\;\text{test}-\;\text{Absorbance}\;\text{of}\;\text{control}\right)}{\text{Absorbance}\;\text{of}\;\text{Control}}\times 100$$

#### 2.12.2. Analgesic activity

To assess the analgesic activity of MNF-TEopt gel, Tail-flick hot water immersion method was employed. The formulation was applied on the dorsal surface of paw to each group. After 15 min treatment of gel, tail of animals (5 cm portion) were immersed into the hot water at 50 ± 5^0^ C. Within few seconds, rats withdrew their tails due to thermal stimulus. To avoid the tail tissue damage, cutoff time of 50 sec was selected. Time when rats withdrew their tail was recorded (Abidin et al., 2016).

#### 2.12.3. Complete Freunds Adjuvant (CFA)-induced anti-arthritis activity

The experiment was performed for 28 days. All the groups except control group (Group S) were injected with 0.1 ml of CFA suspension in sub-plantar region of left hind paw of rat on day 0 (CFA comprised 1 mg/ml heat killed mycobacterium tuberculosis suspended in paraffin oil which engendered inflammatory response within 24 hours). Then, rats were sacrificed after giving 28 days treatment. Parameters such as paw volume, haematological parameters and inflammatory cytokines levels were measured to determine the anti-arthritic activity. Ankle joints were collected after sacrificing the rats and stowed in formalin solution (10%) and stained with dyes (eosin and hematoxylin) for histopathological examination. Radiographical x-ray analysis was performed to confirm the activity (Abidin et al., 2016; Zhang et al., 2021).

## 3. Results and discussion

### 3.1. Optimization of MNF-TE by BBD

**Figure 1.**
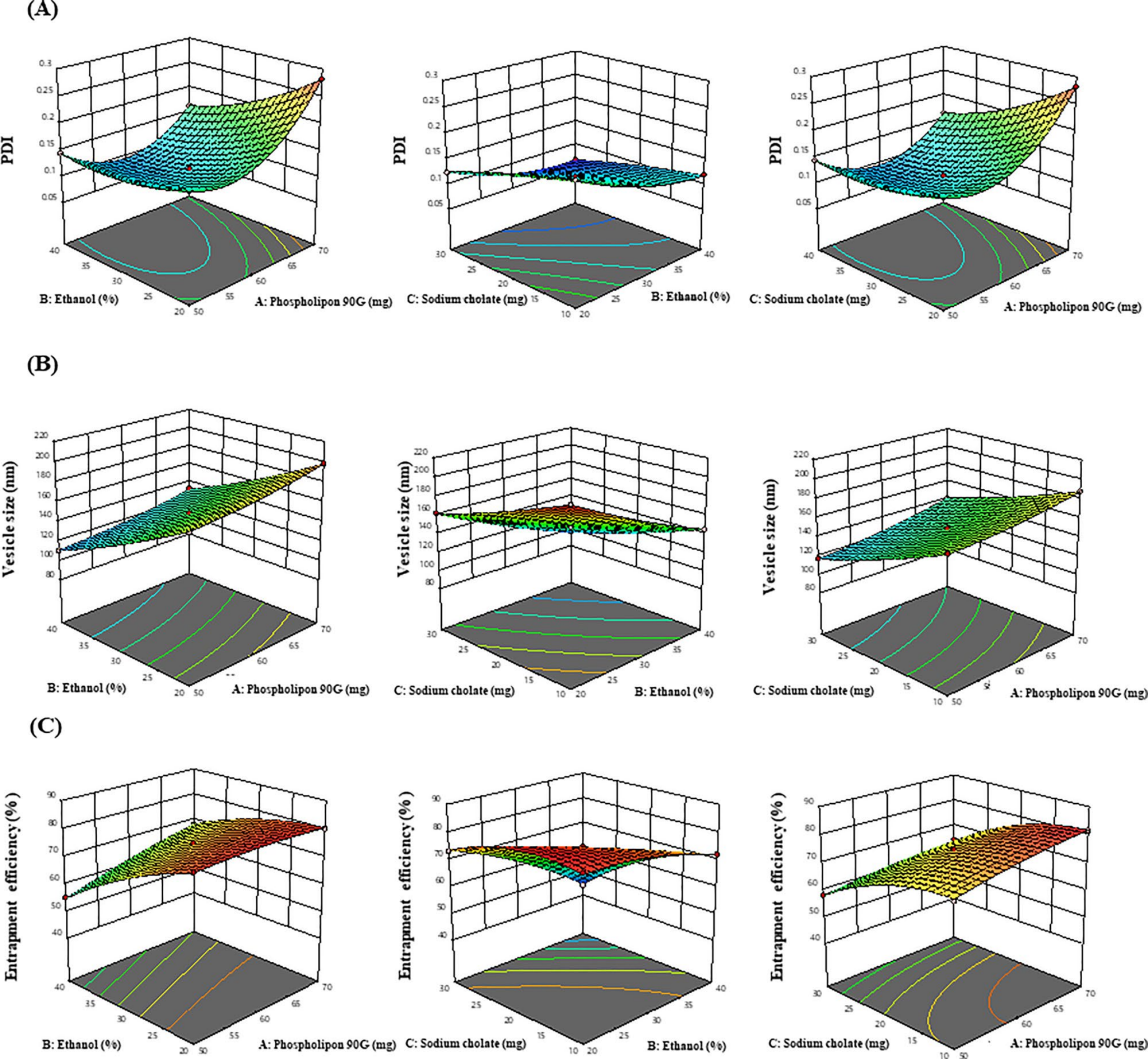
3D-response graphs representing the effect of independent variables on (A) PDI, (B) Vesicle size and (C) Entrapment efficiency.

**Figure 2.**
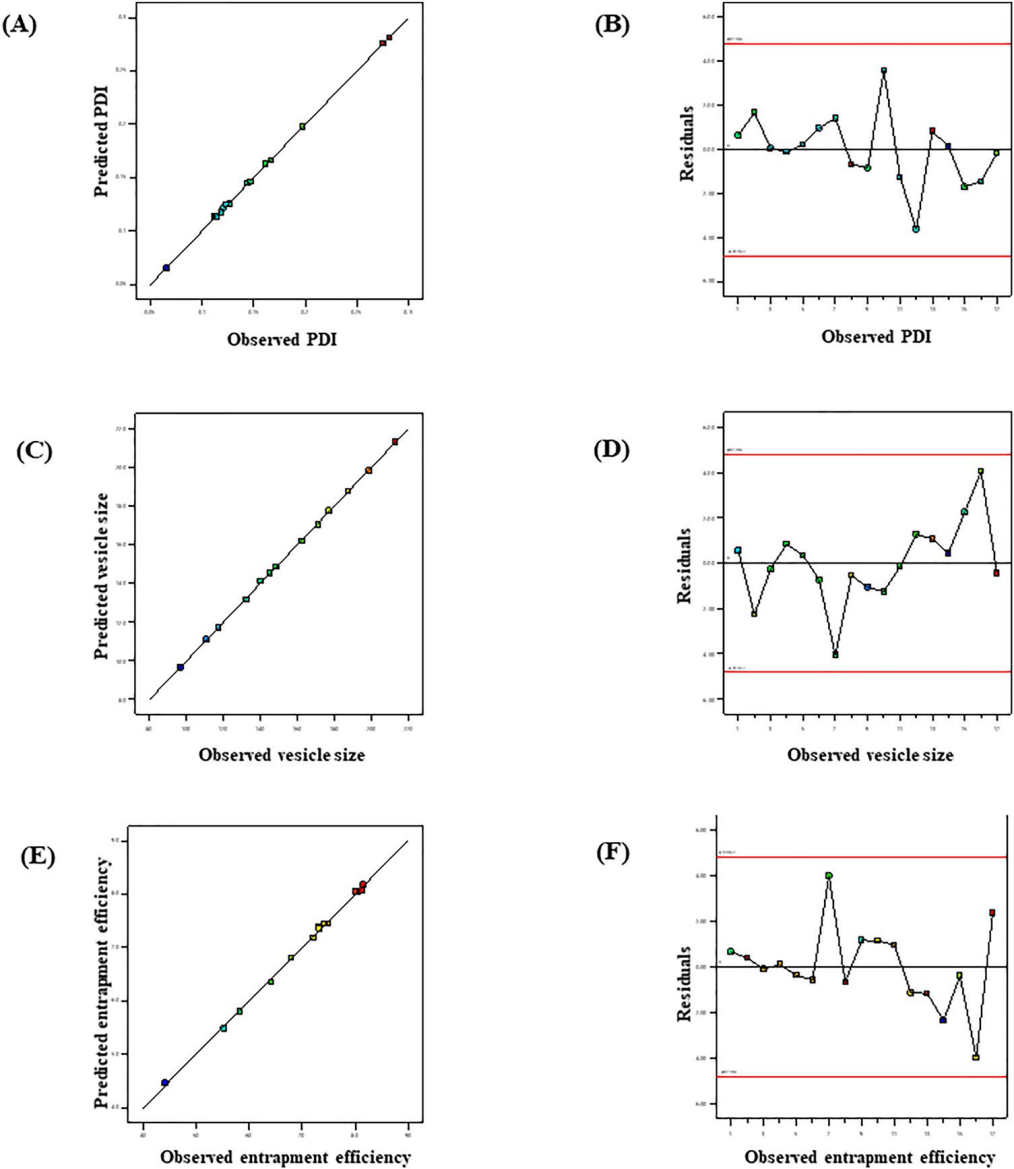
Linear correlation plots (A, C, E) between actual and predicted values and the corresponding residual plots (B, D, F) for various responses.

The impact of adopted parameters (Phospholipon 90 G, ethanol and sodium cholate) on PDI, entrapment efficiency and vesicle size of MNF-loaded transethosomes are evinced by 3D- response diagram illustrated in Figure 1 and the corresponding residual plots for adopted responses and the linear relationship betwixt predicted and experimental values (engendered by BBD) are represented in Figure 2.

#### 3.1.1. Response (1): effect of independent variables on PDI

The PDI of all 17 runs were reckoned to be betwixt 0.0653 and 0.2818 (Table 2).

$$\text{PDI}=+0.1136+0.0333\text{ A }-0.0350\text{ B }-0.0313\text{ C }-0.0243\;\text{AB}\;-0.0437\;\text{AC}+0.0050\;\text{BC}+0{.0580\;A}^{2}+0{.0171\;B}^{2}-0{.0043\;C}^{2}$$

From the above polynomial equation, it was signified that the phospholipon 90 G has a positive effect on PDI. On increasing concentration of phospholipon 90 G (50 to 70 mg), the PDI increased from 0.1265 ± 0.003 to 0.1468 ± 0.007, 0.1670 ± 0.003 to 0.2818 ± 0.012, 0.1442 ± 0.005 to 0.1618 ± 0.004 and 0.1203 ± 0.1209 to 0.2752 ± 0.012 as observed in formulations 7 &1, 2 &13, 9 &15 and 16 &8 respectively. In contrast, ethanol and sodium cholate have negative effect on PDI. Increasing concentration of ethanol from 20 to 40% leads to decreaseed PDI from 0.1670 ± 0.003 to 0.1442 ± 0.005, 0.1237 ± 0.004 to 0.0653 ± 0.002, 0.2818 ± 0.012 to 0.1618 ± 0.004 and 0.1977 ± 0.007 to 0.1191 ± 0.003 as observed in formulations 2 &9, 12 &14, 13 &15 and 17 & 10 respectively. Similarly, increasing sodium cholate concentration from 10 to 30 mg results in decreased PDI from 0.2752 ± 0.012 to 0.1265 ± 0.003, 0.1191 ± 0.003 to 0.0653 ± 0.002, 0.1468 ± 0.008 to 0.1209 ± 0.007 and 0.1977 ± 0.007 to 0.1237 ± 0.004 as observed in formulations 8 &7, 10 &14, 1 &16 and 17 &12 respectively.

#### 3.1.2. Response (2): effect of independent variables on vesicle size

The vesicle size of all 17 runs were reckoned to be betwixt 96.74 and 213.01 (Table 2).

$$\text{Vesicle}\;\text{size}=+148.38+10.29\text{ A – }33.28\text{ B – }25.02\;C+0.0025\;\text{AB}+1.72\;\text{AC}+0.6500\;\text{BC}+2{.97\;A}^{2}+3{.24\;B}^{2}+2{.61\;C}^{2}$$

From experimental data, it was signified that the Phospholipon 90 G has a positive effect on vesicle size. By increasing phospholipon 90 G concentration from (50 to 70 mg), the vesicle size increases from 117.12 ± 4.09 to 140.17 ± 4.03 nm, 176.96 ± 3.92 to 198.51 ± 2.98 nm, 110.65 ± 4.36 to 132.21 ± 2.32 nm and 171.18 ± 3.07 to 187.34 ± 4.26 nm as observed in formulations 1 & 7, 2 & 13, 9 & 15 and 16 & 8 respectively. This could be attributable to the fact that increase in Phospholipon 90 G results in prominent increase of vesicle size. Wherein, ethanol and sodium cholate exhibited the negative impact on vesicle size. By increasing ethanol concentration from (20 to 40%), the vesicle size gets reduced from 176.96 ± 3.92 to 110.65 ± 4.36 nm, 162.25 ± 4.23 to 96.74 ± 2.09 nm, 198.51 ± 2.98 to 132.21 ± 2.32 nm and 213.01 ± 4.35 to 144.9 ± 4.67 nm as observed in formulations 2 & 9, 12 & 14, 13 &15 and 17 & 10 respectively. Similarly, by increasing sodium cholate concentration from (10 to 30 mg), the vesicle size gets reduced from 187.34 ± 4.26 to 140.17 ± 4.03 nm, 144.9 ± 4.67 to 96.74 ± 2.09 nm, 171.18 ± 3.07 to 117.12 ± 4.09 nm and 213.01 ± 4.35 to 162.25 ± 4.23 nm as observed in formulations 8 & 7, 10 & 14, 16 & 1 and 17 & 12 respectively. The decrease in vesicle size by sodium cholate and ethanol could be due to the disruption of bilayer structure of cellular membrane beyond their certain concentration.

#### 3.1.3. Response (3): effect of independent variables on entrapment efficiency

From experimental data, it was observed that independent variables have significant effect on entrapment efficiency (EE) and the EE of all 17 runs were found to be betwixt 44.14 and 81.55% (Table 2).

$$\text{Entrapment}\;\text{efficiency}=+74.44+3.35\text{ A }-9.45\text{ B }-8.54\;C+3.28\;\text{AB}\;-0.05775\;\text{AC}\;-4.98\;\text{BC}+0{.9483\;A}^{2}-2{.56\;B}^{2}-4{.20C}^{2}$$

From the above polynomial equation, it was signified that Phospholipon 90 G has positive effect on entrapment efficiency wherein, ethanol and sodium cholate have negative effect on entrapment efficiency. It was observed that an increment in Phospholipon 90 G concentration (50 to 70 mg) improve the entrapment efficiency from 58.2 ± 1.34 to 64.22 ± 2.98%, 80.10 ± 1.72 to 80.45 ± 1.23%, 55.21 ± 2.09 to 67.98 ± 1.62% and 73.22 ± 1.92 to 81.55 ± 1.68% as observed in formulations 1 &7, 3 & 2, 9 & 15, 16 & 8 respectively. This could be due to the expansion of bilayer domain dimension resulting from formation of more number of TE vesicles which provides more space for MNF entrapment in TE vesicles.

From the experimental data, it can be signified that the increase in ethanol concentration (20 to 40%) results in decreased entrapment efficiency of MNF in TE vesicles from 80.45 ± 1.68 to 55.21 ± 1.72%, 73.23 ± 1.62 to 44.14 ± 1.76%, 80.1 ± 1.92 to 60.98 ± 1.23% and 81.26 ± 1.98 to 72.11 ± 1.87% as observed in formulations 2 & 9, 12 & 14, 13 & 15 and 17 & 10 respectively. Similarly, increase in sodium cholate concentration (10 to 30 mg) results in decreased entrapment efficiency from 81.55 ± 1.62 to 64.22 ± 2.98%, 72.11 ± 1.87 to 44.14 ± 1.76%, 73.22 ± 2.09 to 58.2 ± 1.34% and 81.26 ± 1.98 to 73.23 ± 1.62% as observed in formulations 8 & 7, 10 & 14, 16 & 1 and 17 & 12 respectively. This could be due to the fact that beyond a certain concentration, ethanol and sodium cholate disrupts the vesicular bilayered membrane structure which leads to loss of drug from the TE vesicles.

Based on the above experimental results, optimized formulation was prepared with phospholipon 90 G (60 mg), ethanol (30%) and sodium cholate (20 mg) as per the formula generated by the point prediction method and further evaluated for PDI, entrapment efficiency and vesicle size. The MNF-TEopt exhibited the vesicle size of 146.8 ± 2.98 nm, entrapment efficiency of 74.23 ± 2.09% and PDI of 0.1139 ± 0.009 which were in proximity with the predicted values of PDI of 0.1136, vesicle size of 148.38 nm, and entrapment efficiency of 74.44% engendered by the Box-Behnken design.

### 3.2. Characterization

**Figure 3.**
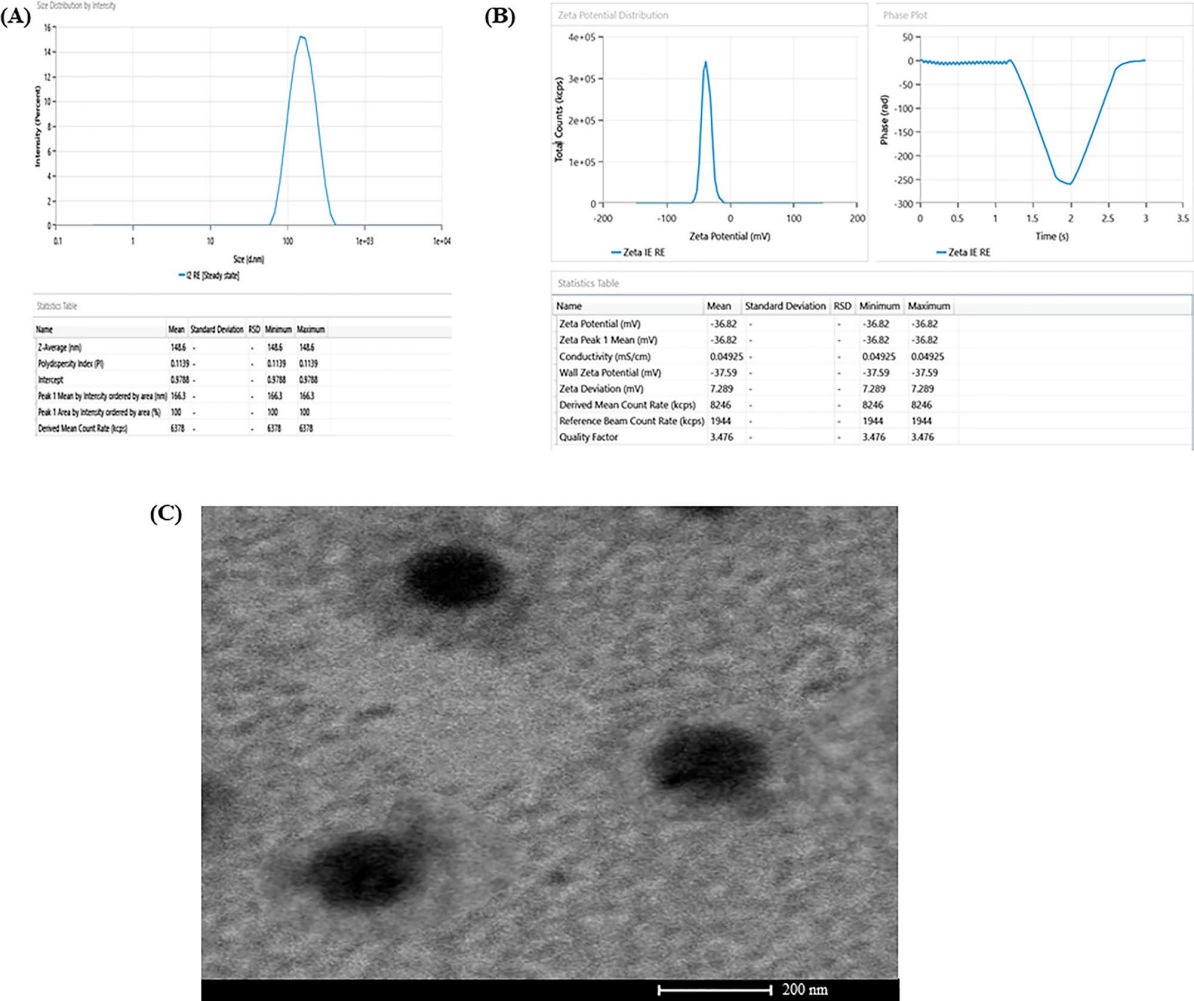
(A) Vesicle size distribution of MNF-TEopt formulation, (B) Zeta-potential of MNF-TEopt formulation and (C) Transmission electron microscopy of MNF-TEopt formulation.

Average vesicle size and PDI of MNF-TEopt was experimentally reckoned to be 146.8 nm and 0.1139 respectively (Figure 3A) with entrapment efficiency of 74.23% wherein the predicted values of average vesicle size and PDI were 148.38 nm and 0.1136 with entrapment efficiency of 74.44%. The experimentally observed values of all responses were in proximity with the predicted values confirming the legitimacy and consistency of the model. Furthermore, the zeta potential of MNF-TEopt was reckoned to be −38.62 mV (Figure 3B).

### 3.3. Transethosome Morphology

The TEM analysis of MNF-TEopt formulation unveiled that the prepared vesicles were spherical in shape with well-defined sealed structure and uniform size distribution (Figure 3C).

### 3.4. Texture analysis

**Figure 4.**
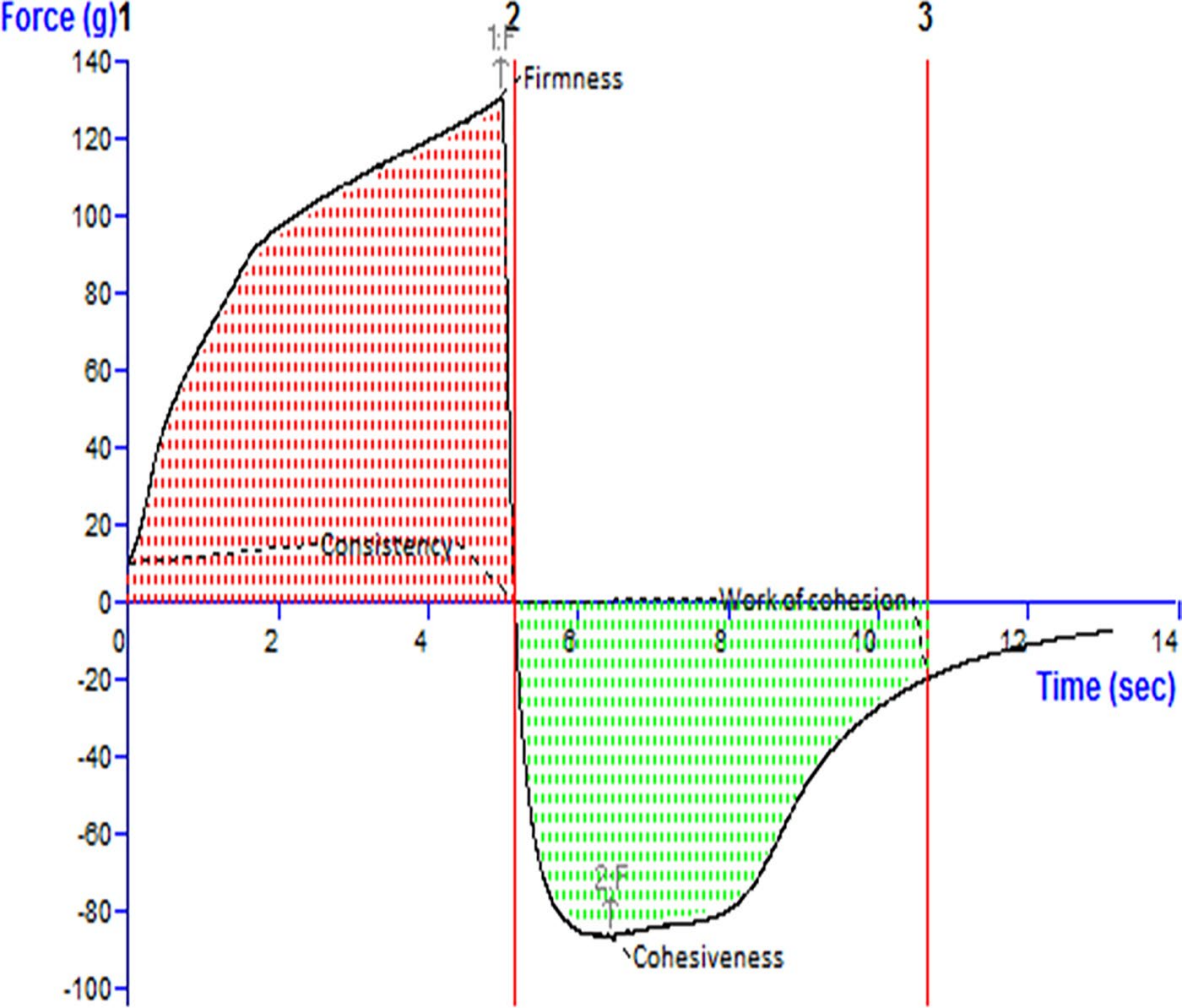
Texture analysis diagram of MNF-TEopt gel showing firmness, consistency, cohesiveness and work of cohesion.

The texture analysis of MNF-TEopt gel is illustrated in Figure 4. The firmness, consistency, cohesiveness and work of cohesion of MNF-TEopt were reckoned to be 145.35 g, 501.56 g.sec, −90.77 g and −357.59 g.sec respectively (Figure 4).

### 3.5. In vitro drug release study

**Figure 5.**
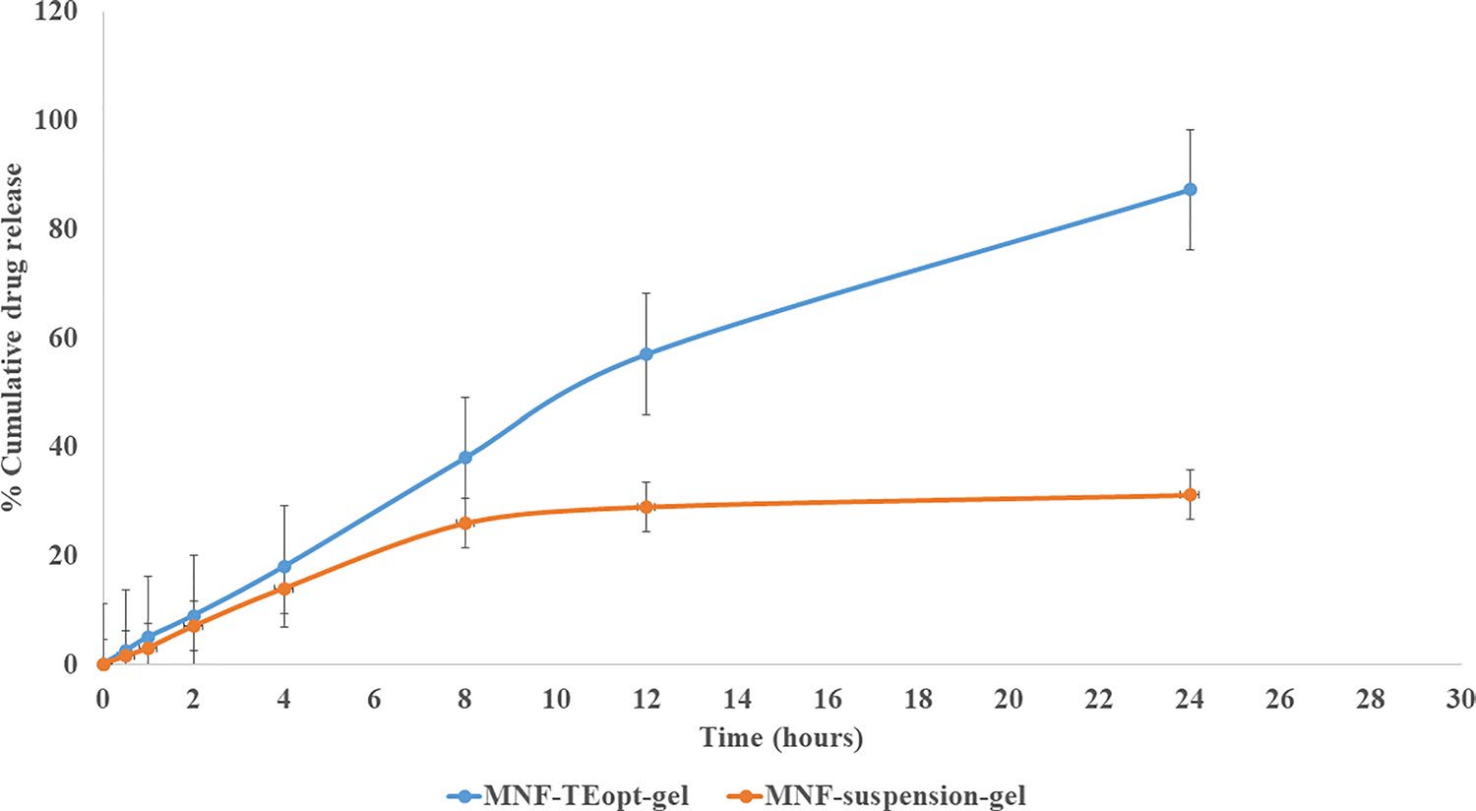
Comparative in vitro drug release profile of MNF suspension gel and MNF-TEopt gel.

The in vitro release of mangiferin from MNF suspension through dialysis membrane was reckoned to be 31.19% wherein the optimized MNF-TEopt formulation presented 87.32% release of mangiferin through dialysis membrane (Figure 5). Data acquired from in-vitro drug release experiment was fitted into diverse mathematical kinetics model (first-order, zero-order, korsmeyer peppas, and Higuchi kinetics model) and out of which, Higuchi kinetics model revealed the maximum R^2^ value illustrated in Table 3. Ergo, it can be stated that the release of mangiferin from MNF-TEopt gel follows a higuchi diffusion mechanism.

**Table 3.** Invitro drug release kinetics with correlation values.

Release Kinetics	R^2^	Equation	X-axis	Y-axis
Korsmeyer-Peppas	0.962	M_t_/M∞ = K_tn_	Log fraction of drug released	Log time
Higuchi	0.979	M_t_ = M_0_ + k_h_t_1/2_	Fraction of drug released	√time
Zero-order release	0.951	M_t_ = M_0_ + k_0_ t	Fraction of drug released	time
First-order release	0.946	ln M_t_ = ln M_0_ + k_1_ t	Log % drug remaining	time

### 3.6. Skin permeation study

**Figure 6.**
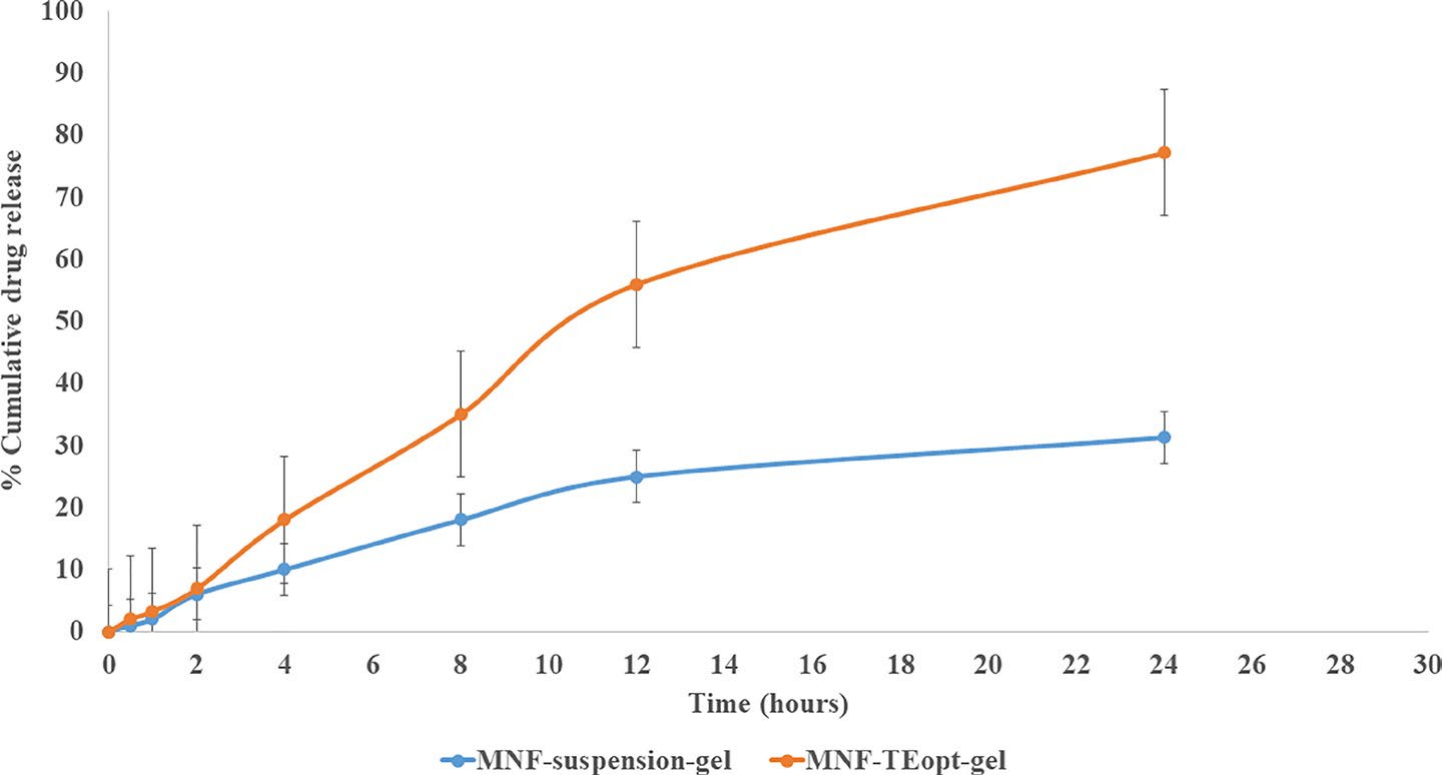
Comparative in vitro skin permeation study of MNF suspension gel and MNF-TEopt gel across rat skin.

The skin permeation study revealed the permeation of 31.29% of mangiferin from MNF suspension gel with transdermal flux value of 1.77 µg/cm^2^/h wherein the MNF-TEopt formulation exhibited permeation of 77.21% mangiferin through the rat skin with transdermal flux value of 4.7 µg/cm^2^/h (Figure 6).

### 3.7. Confocal Laser Scanning microscopy

**Figure 7.**
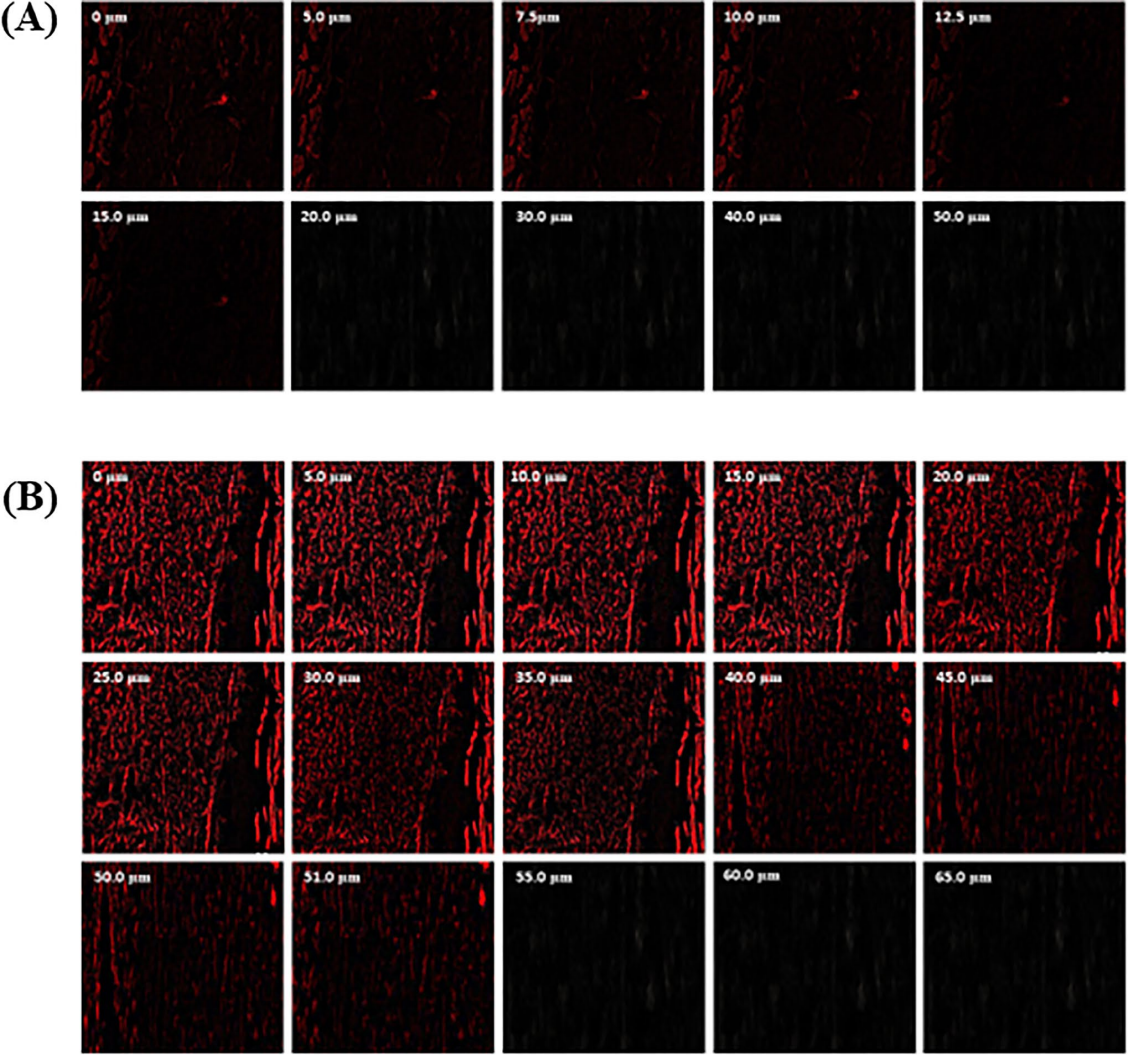
CLSM images in optical cross section perpendicular to rat skin surface (A) treated with MNF suspension gel and (B) treated with MNF-TEopt gel formulation.

The CLSM analysis revealed that the MNF-TE formulation penetrated deeper into the layer of skin (upto 52 um) in comparison with the MNF suspension (control) which was confined to depth of 15 um perse (Figure 7).

### 3.8. Skin irritation study

**Figure 8.**
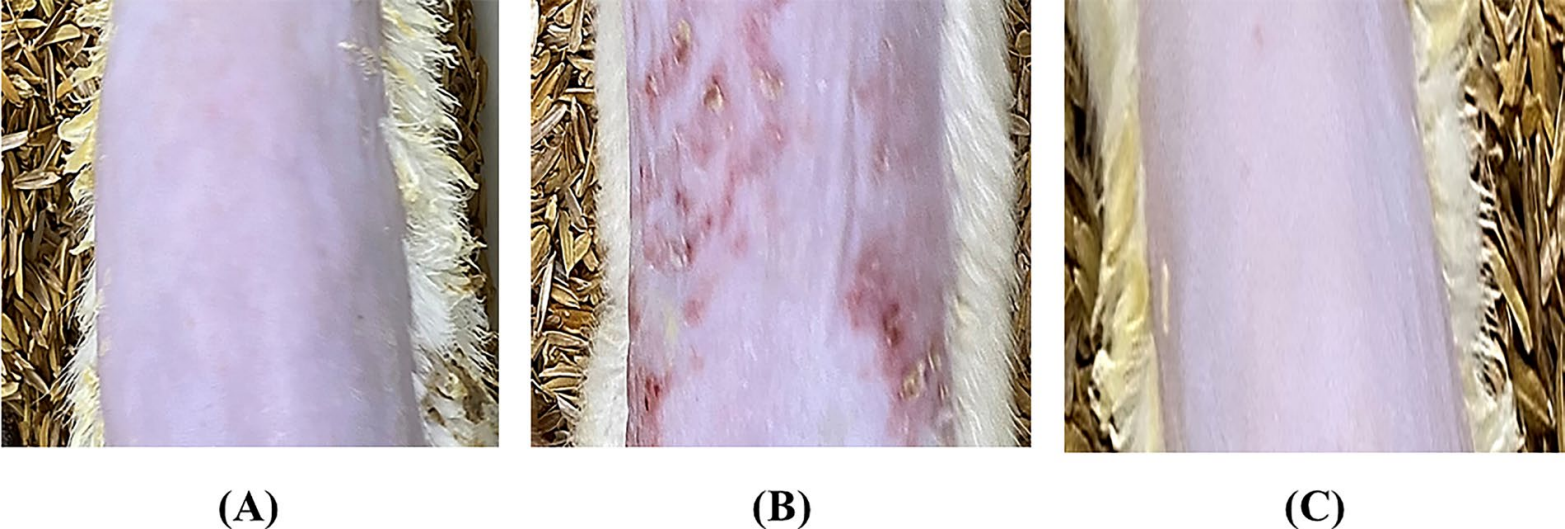
Skin irritation images of rat treated with (A) Normal control, (B) Positive control and (C) MNF-TEopt gel.

The skin irritation study of MNF-TE gel was performed on Wistar albino rats (Figure 8) and compared with the formalin solution (skin irritant and positive control) and the scores of edema and erythema of treated groups were observed which are tabulated in Table 4. The skin treated with formalin solution exhibited high irritation scale of edema (3.4 ± 0.7) and erythema (2.3 ± 0.4) respectively, wherein the MNF-TE gel exhibited very less irritation with irritation scale of edema (0.4 ± 0.02) and erythema (0.3 ± 0.01). The MNF-TE gel treated skin espied absence of inflammation and redness. The results confirmed that the MNF-TE gel formulation is nonirritant.

**Table 4.** Draize irritation score after application of MNF-TE gel and formalin solution on wistar albino rats.

	Positive control		MNF-TE gel	
Rat	Edema	Erythema	Edema	Erythema
1	4	4	0	0
2	3	3	1	0
3	3	4	0	0
**Mean ± SD**	3.33 ± 0.31	3.66 ± 0.42	0.33 ± 0.12	0 ± 0

### 3.9. Pharmacokinetic study

**Figure 9.**
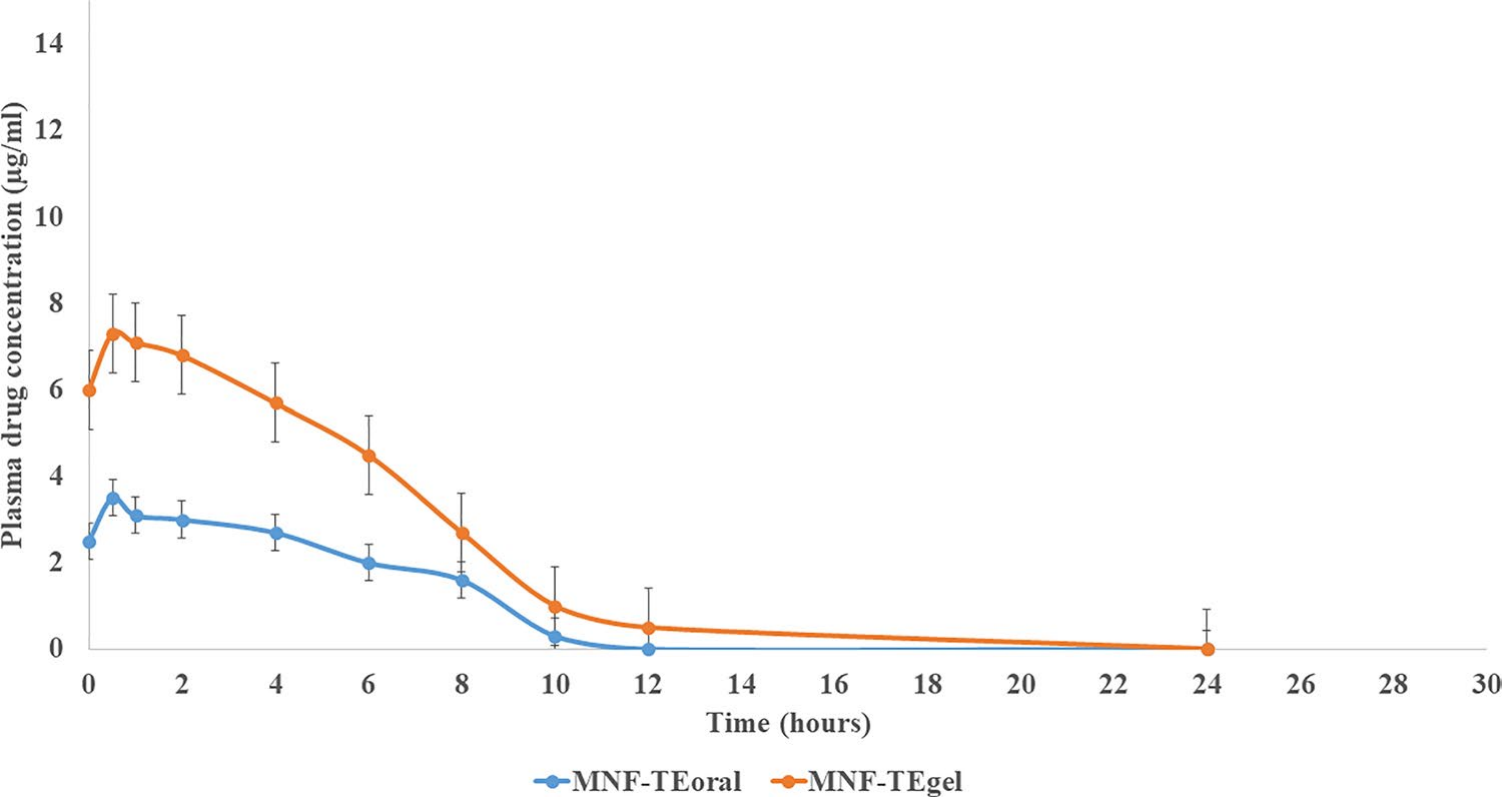
Plasma drug concentration profiles of mangiferin after oral and transdermal delivery.

To assess the in vivo behavior of MNF-TE gel, pharmacokinetic studies were performed on rats. The concentration of MNF in plasma after oral administration of MNF-TE formulation and transdermally application of MNF-TE gel was measured by HPLC (HPLC chromatograms are shown in Supplementary material) using reported method and results are illustrated in Figure 9 and Table 5. C_max_ and AUC_0-24h_ of the MNF-TE formulation after oral administration were 3.74 ± 1.91 μg/ml and 22.96 ± 9.76 μg.h/ml respectively which were significantly lower than those of (*p* < 0.01) transdermally applied MNF-TE gel (6.94 ± 0.51 μg/ml and 43.92 ± 7.90 μg.h/ml respectively) revealing that absorbtion of MNF was potentially increased on transdermally application of MNF-TE gel.

**Table 5.** Pharmacokinetic parameters.

Pharmacokinetic Parameters	MNF-TE oral	MNF-TE gel
**C_max_**	3.74 ± 1.91 ug/ml	6.94 ± 0.51 ug/ml
**T_max_**	0.5 h	0.5 h
**T_1/2_**	5.16 h	10.42 h
**AUC_0-24h_**	22.96 ± 9.76 ug.h/ml	43.92 ± 7.90 ug.h/ml

### 3.10. Anti-arthritic activity

#### 3.10.1. In vitro antiarthritic activity

Protein denaturation is one of the manifestation of rheumatoid arthritis which results in autoantigens production. In vitro antiarthritic activity of MNF-TE formulation was assessed by protein denaturation method in which diclofenac gel was used as standard gel for comparison. The results unveiled that the MNF-TE gel exhibited percent inhibition of 81.28% in inflammation while standard gel showed the percent inhibition of 78.28% which implies that developed formulation has better antiarthritic potential than marketed gel.

#### 3.10.2. Analgesic activity

The results attained from analgesic study on wistar albino rats unveiled that the animals treated with control formulation presented thermal stimulus time of 22.53 sec wherein animals treated with CFA (toxic group) showed thermal stimulus time of 13.57 sec. The animals treated with diclofenac gel (standard) showed thermal stimulus time of 25.36 sec and the animals treated with MNF-TE gel presented the thermal stimulus time of 30.23 sec which was higher than diclofenac standard gel (*p* < 0.05). It can be stipulated that the increase activity by MNF-TE gel can be attributable to higher permeation of MNF into skin layers than the control and standard and thus it has higher analgesic activity.

#### 3.10.3. Cfa- induced anti-arthritic model

After 28 days of treatment with MNF-TE gel, biochemical analysis and paw volume measurement were performed onrats to evaluate the anti-arthritic activity. The radiographic analysis were carried out to corroborate the anti-arthritic activity.

##### 3.10.3.1. Paw volume

**Figure 10.**
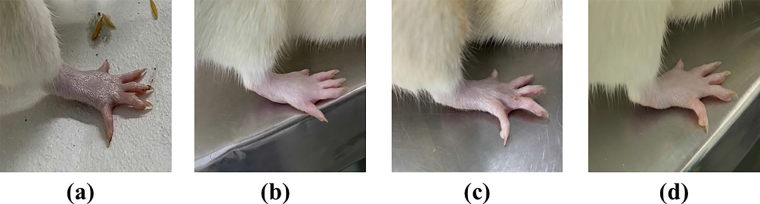
Images showing paw edema of rats under different treatment (A) Control, (B) CFA (Toxic control), (C) Diclofenac gel and (D) MNF-TEopt gel.

The paw volume was measured on 0, 7^th^, 14^th^ and 28^th^ day from initiation of experiment. Dunnett’s test for statistical analysis were appertained to the results acquired with respect to paw volume. The results unveiled that from day 0-7, there was increase in paw volume in all animal groups. MNF-TE gel revealed the significant decrease in paw volume in comparison with the diclofenac standard gel which signifies the therapeutic potential of MNF-TEopt gel wherein toxic group which received no treatment displayed persistent increase in paw volume (Table 6 and Figure 10).

**Table 6.** Effect of different treatments on paw edema of rats.

Paw volume					
Treatment	Day 0	Day 7	Day 14	Day 21	Day 28
Gel base (control)	1.25 ± 0.07	1.28 ± 0.04	1.31 ± 0.03	1.30 ± 0.02	1.31 ± 0.03
CFA (toxic control)	1.45 ± 0.03	1.92 ± 0.02	2.18 ± 0.04	2.26 ± 0.03	2.31 ± 0.06
CFA + diclofenac gel	1.54 ± 0.07	1.77 ± 0.03	1.72 ± 0.02	1.68 ± 0.04	1.64 ± 0.04
CFA + MNF-TE gel	1.47 ± 0.04	1.71 ± 0.02	1.62 ± 0.03	1.56 ± 0.02	1.50 ± 0.02

##### 3.10.3.2. Haematological parameters

From previously published reports, it was observed that in rheumatoid arthritis due to anemia, RBC count decreases significantly attributable to abnormal storage of Iron (Fe) in synovial tissue and reticuloendothelial system and the bone marrow failure in anemia wherein WBC count and ESR increases ascribable to stimulation of immune cells by body in presence of invading antigens. After 28 days of treatment, RBC, WBC and ESR counts were assessed on 29^th^ day (Table 7). From experimental results, it was observed that RBC count dwindled in toxic group wherein MNF-TE gel and standard gel exhibited elevation in RBC count but lesser in comparison with control group. Also, the MNF-TE gel and standard marketed gel showed nearly same RBC count but not higher than that of toxic group implying good antiarthritic potential. Similarly, WBC count and ESR were reckoned to be lower than that of toxic group stipulating lesser inflammation than group treated with diclofenac standard gel.

**Table 7.** Effect of different treatments on haematological parameters.

Treatment	RBC count (mm^3^)	WBC count (mm^3^)	ESR (mm/h)
Gel base (control)	7.35 ± 0.07	6524 ± 68.4	2.3 ± 0.3
CFA (toxic control)	5.18 ± 0.03	7697 ± 22.2	2. 8 ± 0.21
CFA + diclofenac gel	6.51 ± 0.07	7098 ± 11.3	2.5 ± 0.22
CFA + MNF-TE gel	6.97 ± 0.04	6726 ± 23.2	2.48 ± 0.33

To assess the anti-inflammatory effects of MNF-TE gel, IL-1β, TNF-α and IL-6 levels were measured in blood. All inflammatory cytokines showed markedly decrease in their levels in MNF-TE gel group relative to those in standard diclofenac gel group and control group which could be attributable to the higher permeation of MNF-TE gel in skin layers (Table 8).

**Table 8.** Effect of different treatments on inflammatory cytokines.

Treatment	TNF- α( pg/ml)	IL-1β(pg/ml)	IL-6 (pg/ml)
Gel base (control)	600 ± 10.7	18.7 ± 0.4	41 ± 1.3
CFA (toxic control)	1812 ± 21.03	80.2 ± 1.2	227 ± 5.21
CFA + diclofenac gel	1294 ± 27.07	37.8 ± 0.8	105 ± 7.2
CFA + MNF-TE gel	702 ± 17.4	21.9 ± 0.9	54 ± 6.3

##### 3.10.3.3. Histological examination

**Figure 11.**
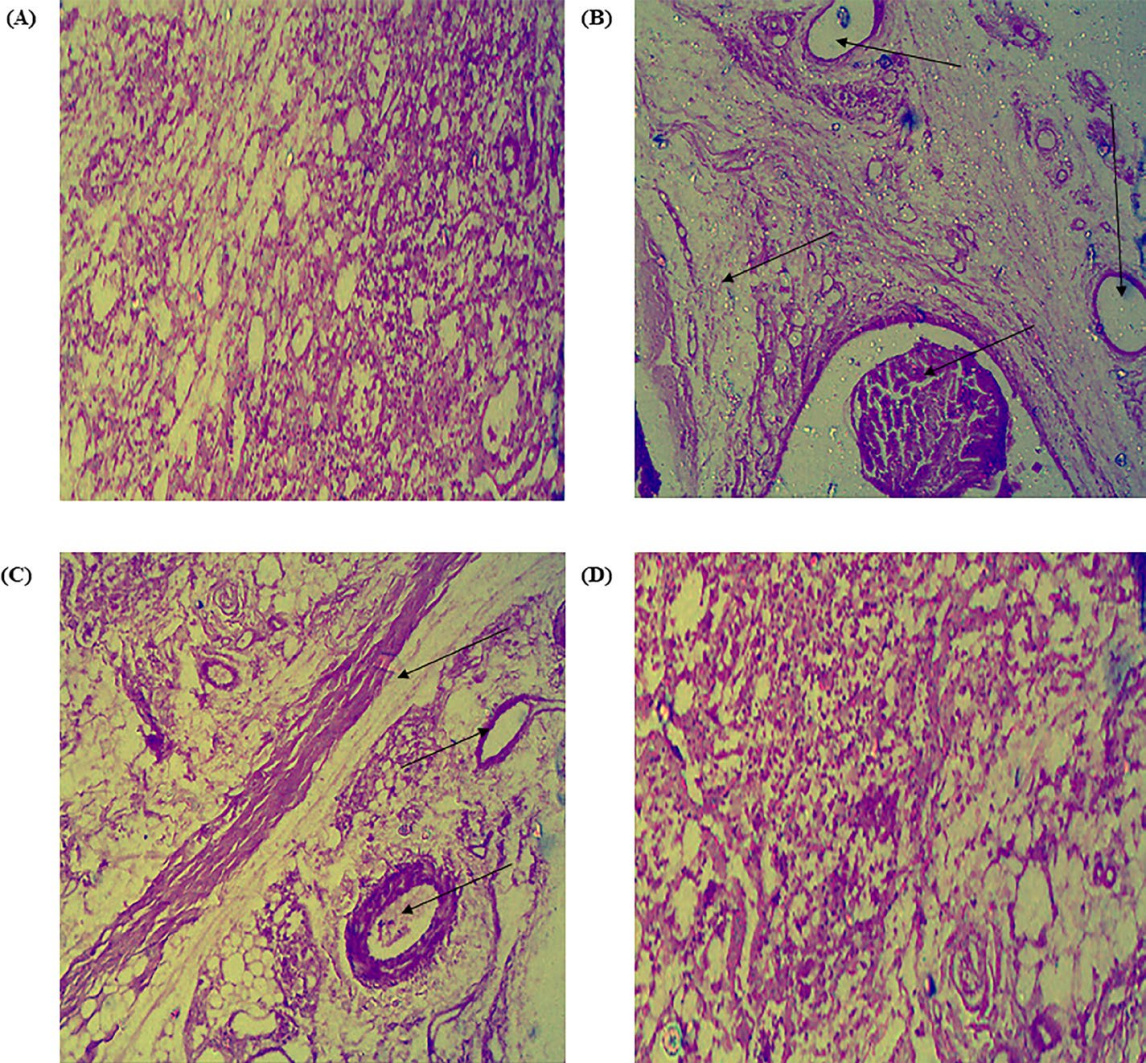
Histopathological images of rat ankle joint tissues stained (lower row) with hematoxylin and eosin under different treatment (A) Control, (B) CFA (Toxic control), (C) Diclofenac gel and (D) MNF-TEopt gel. Arrows show inflammatory cell infiltration.

Histological examination of the hind paws were performed which revealed that the joints of the rats in toxic group exhibited severe synovial damage and large number of inflammatory cell infiltration. The rats treated with standard diclofenac gel showed the occasional synovial damage and less inflammatory cell infiltration. In contrast, the group treated with MNF-TE gel exhibited the most extensive protection from synovial damage and least inflammatory cell infiltration among the other groups (Figure 11).

##### 3.10.3.4. Radiographic analysis

**Figure 12.**
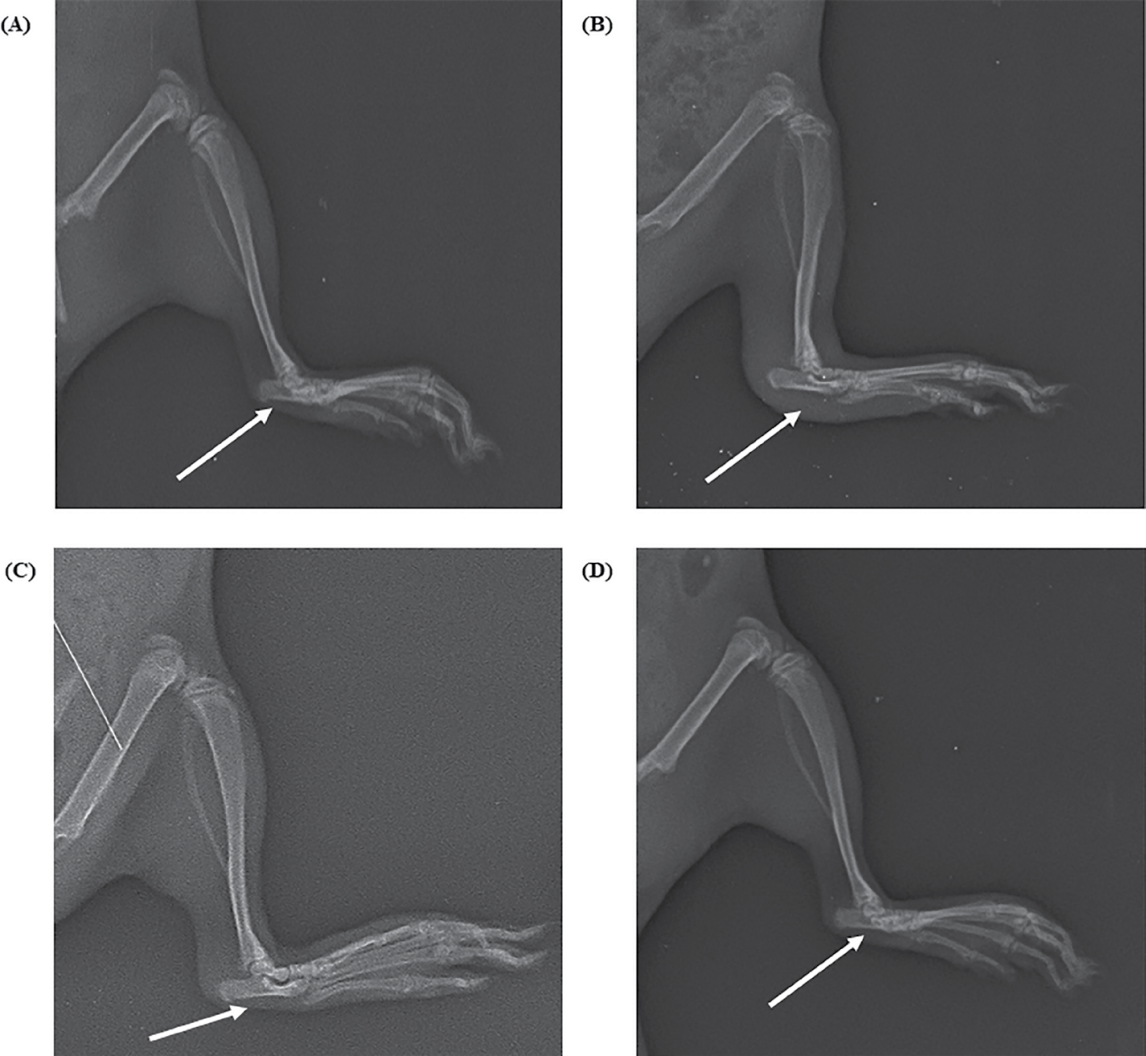
X-ray of different groups of rats under different treatment (A) Control, (B) CFA (Toxic control), (C) Diclofenac gel and (D) MNF-TEopt gel.

X-rays of hind paws of all animal groups were performed for assessing bone damage (Figure 12) in which (Figure 12a) shows the control group with no arthritic condition, (Figure 12b) shows the toxic group which received no treatment after induction of inflammation (the arrow signifies the inflamed joint and severity of arthritis exhibited by degree of whiteness) and (Figure12 c and 12d) shows the group treated with MNF-TE gel and diclofenac marketed gel in which the arrow signifies the inflamed joint with lesser arthritic state and good recovery. Figure 12c reveals more recovery than Figure 12d due to high permeation of MNF-TE gel into the skin layers and good therapeutic potential of MNF in rheumatoid arthritis.

## 4. Conclusion

The current research comprehends the development and optimization of MNF-loaded TE formulation by utilizing BBD which engendered the better anti-rheumatic activity via transdermal route. The elite MNF-TE formulation manifested nano size of 148.6 nm, EE of 74.23% with PDI of 0.1139. The CLSM study unveiled that the developed MNF-TE formulation has greater permeation of MNF across the skin layers in comparison with the MNF suspension gel and a greater in-vitro release withal. The pharmacokinetic study demonstrated significant levels of MNF in blood via transdermal route in comparison with the oral administration. The in vivo study unveiled that the MNF-TE formulation manifested enhanced analgesic effect than the standard marketed gel. Biochemical analysis showed suppression in inflammatory cytokines (IL-1β, TNF-α and IL-6), dwindled WBC count and enhanced RBC count. Rat paw volume indicates a significant consistent decrease in the paw edema. From the radiographic analysis, it was depicted that the group treated with MNF-TE formulation exhibit higher recovery phase than the standard marketed gel. The outcomes corroborate that the prepared TE vesicle formulation is a treasured carrier for the MNF transdermal delivery for the management of rheumatoid arthritis.

## Ethical approval

The animal study protocol was permitted by the Institutional Animal Ethical Committee (IAEC), Jamia Hamdard, New Delhi, India) with the approved animal study protocol 173/Go/Re/S/2000/CPCSEA (Approval No. 1847, 2022). The current study was performed following the guidelines of the Declaration of Helsinki.

## Supplementary Material

Supplemental Material
