# Supplementary material for: Nanotransethosomes for enhanced transdermal delivery of mangiferin against rheumatoid arthritis: formulation, characterization, invivo pharmacokinetic and pharmacodynamic evaluation
Source: Drug Deliv. 2023 Feb 2;30(1):2173338. doi: 10.1080/10717544.2023.2173338 (PMC9943251; doi:10.1080/10717544.2023.2173338)
Supplement: Supplemental Material [file IDRD_A_2173338_SM3885.docx]

**Supplementary material**: HPLC chromatograms for Pharmcokinetic study


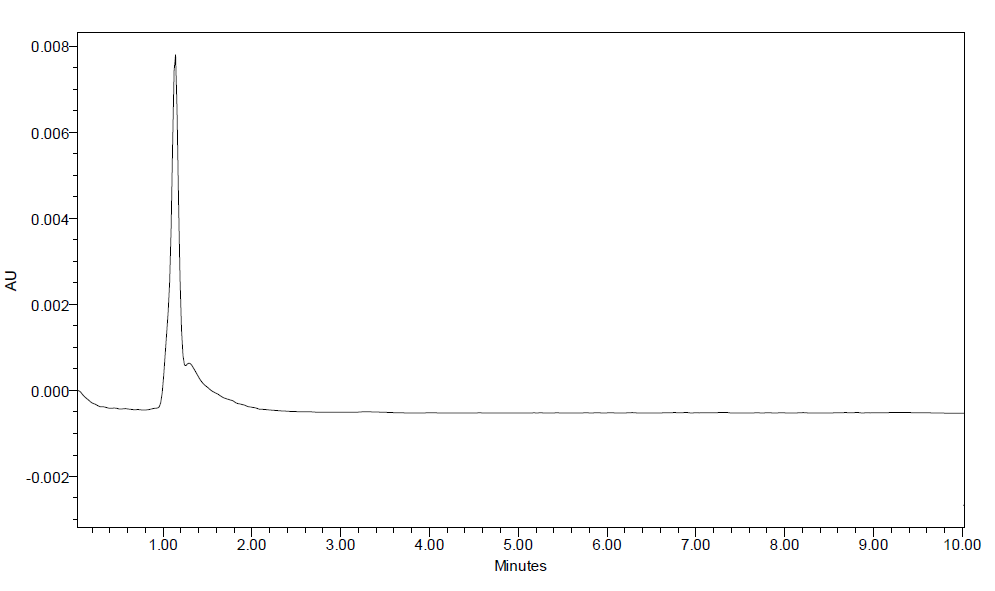


Fig. S1 : HPLC chromatogram of Blank in Plasma


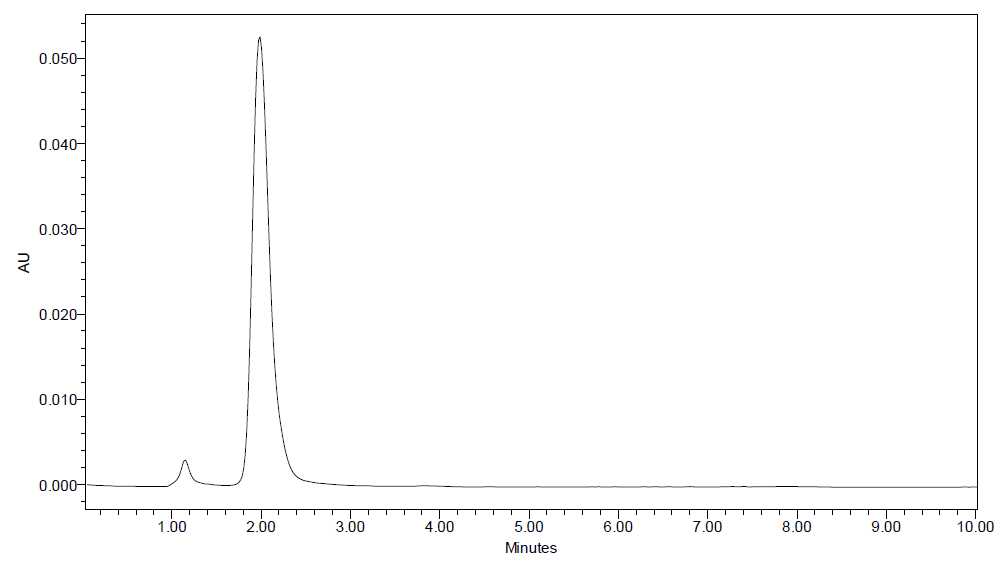
Fig. S2: HPLC chromatogram of Standard mangiferin in Plasma


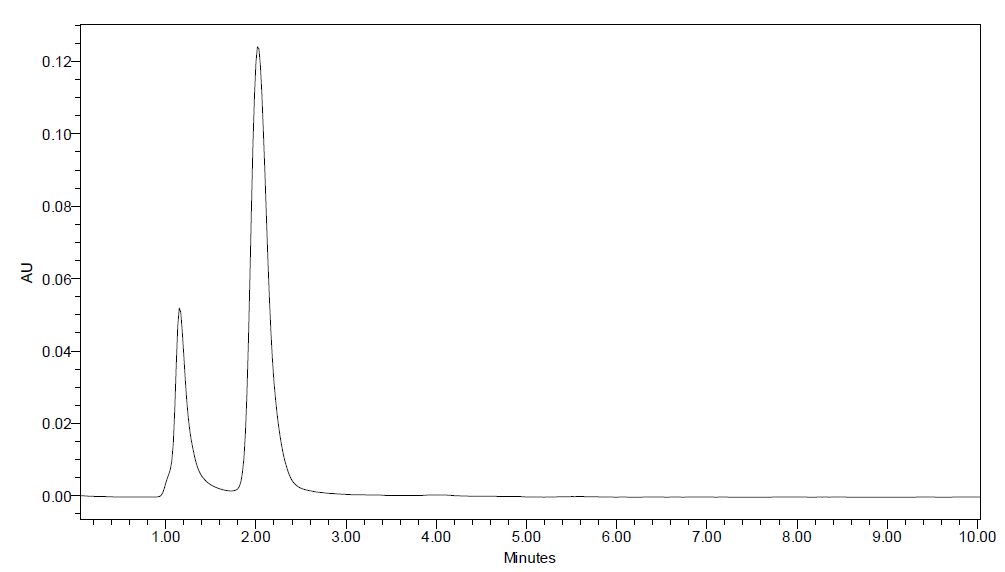


Fig. S3: HPLC chromatogram of MNF-TEopt gel in Plasma
